# Three‐dimensional evaluation of morphology and position of impacted supernumerary teeth in cases of cleidocranial dysplasia

**DOI:** 10.1111/cga.12358

**Published:** 2019-10-23

**Authors:** Michiko Tsuji, Hiroyuki Suzuki, Shoichi Suzuki, Keiji Moriyama

**Affiliations:** ^1^ Maxillofacial Orthognathics, Department of Maxillofacial Reconstruction and Function, Division of Maxillofacial/Neck Reconstruction, Graduate School of Medical and Dental Sciences Tokyo Medical and Dental University Tokyo Japan

**Keywords:** cleidocranial dysplasia, cone‐beam computed tomography (CBCT), supernumerary teeth, three‐dimensional (3D) image analysis

## Abstract

Cleidocranial dysplasia (CCD) is a congenital anomaly characterized by the presence of impacted supernumerary teeth and delayed eruption of permanent teeth. However, there has been no detailed investigation on supernumerary teeth in patients with CCD using three‐dimensional (3D) imaging techniques. The purpose of this study was to elucidate the morphology and position of supernumerary teeth using 3D images reconstructed from cone‐beam computed tomography (CBCT) data in a group of five Japanese subjects (male, 3; female, 2; age, 15.0‐25.4 years) with CCD. All five subjects exhibited supernumerary teeth (39 in total; average, 7.8; range, 1‐15). All supernumerary teeth were impacted and existed as pairs with adjacent permanent teeth. Comparison of the size (the crown and dental‐root lengths, the crown mesiodistal and buccolingual diameters), the number of cusps and dental roots, the position, and direction of supernumerary teeth in relation to the adjacent permanent teeth was analyzed. The results of relationship analyses revealed that, at sites other than the molar region, supernumerary teeth were positioned on the lingual and distal sides and supernumerary teeth resembled the morphology of their adjacent permanent teeth in terms of the number of cusps but were smaller than the adjacent permanent teeth. In the molar region, supernumerary teeth were microdontia, which were apparently small and obscure morphologically. In addition, while all adjacent permanent teeth exhibited normal direction, five supernumerary teeth exhibited inverse direction. The findings of this study will improve our understanding of the characteristics of CCD and provide important information for the pathophysiology and clinical treatment.

## 1 INTRODUCTION

Cleidocranial dysplasia (CCD; OMIM 119600) is an autosomal dominant inheritance disorder with skeletal characteristics including patent sutures and/or fontanels, hypoplastic or aplastic clavicles, wormian bone formation, and short stature.^1^, ^2^, ^3^, ^4^ Mutations in the runt‐related transcription factor 2 gene (*RUNX2*; core‐binding factor alpha 1 gene [*CBFA1*]), located on chromosome 6p21, have been identified as being responsible for CCD.^5^, ^6^ The incidence of CCD is estimated to be 1 in 1 000 000 cases.^7^ While familial cases of CCD are consistent with autosomal dominant inheritance with marked phenotypic variability, many cases of this disorder appear to be sporadic.^8^ Phenotypic characteristics in the oral cavity include the presence of impacted teeth such as supernumerary teeth, delayed eruption of permanent teeth, and prolonged retention of primary teeth.^9^, ^10^, ^11^ Because supernumerary teeth are frequently accompanied by serious problems in occlusion, dentition, and mastication, such as delayed eruption and impaction of permanent teeth, patients with CCD require comprehensive surgical and orthodontic intervention, such as extraction of supernumerary teeth that disturbed the eruption of adjacent permanent teeth and teeth‐eruption guidance as fenestration‐traction of completely impacted permanent teeth.^12^, ^13^

In general, supernumerary teeth occur in 6% or more of the normal population^14^ and they are etiologically heterogeneous and highly variable, differing in number, position, morphology, and status in primary and/or permanent dentition.^15^, ^16^ While supernumerary teeth are idiopathic in most cases, multiple supernumerary teeth are rare and can be associated with syndromic diseases other than CCD, such as Gardner's syndrome.^17^, ^18^

Retrospective studies have evaluated the number, morphology, position, and status of supernumerary teeth in non‐syndromic cases using extracted teeth or two‐dimensional (2D) imaging techniques such as panoramic radiography.^19^, ^20^, ^21^ Supernumerary teeth are classified on the basis of morphology or location in dental arches. Cases involving one or two supernumerary teeth most commonly involve the anterior maxilla, and the most frequently identified supernumerary teeth are mesiodens (maxillary anterior incisor region), followed by supernumerary teeth in the mandibular premolar region. The morphology of supernumerary teeth presenting in primary dentition is usually normal or conical, while that in permanent dentition is more variable.^19^, ^20^, ^21^, ^22^

In patients with CCD, previous studies have reported the number of supernumerary teeth to range from 1 to 21 (average, 8)^9^ and 0 to 15 (average, 7.5),^12^ with the more frequent locations being the maxillary incisor (22.2%) and mandibular premolar (14.7%) regions.^9^ Studies have also demonstrated a wide variation in supernumerary‐tooth formation in individuals with CCD bearing identical gene mutations.^23^ Abnormalities in tooth morphology and position lingually, identified using panoramic, intraoral, and cephalometric radiographs, and surgically extracted teeth have been reported.^12^

Recent techniques for three‐dimensional (3D) imaging of the craniofacial region using cone‐beam computed tomography (CBCT) have made imaging of dentomaxillofacial structures more practical.^24^, ^25^ CBCT is a useful tool not only for accurately determining the morphology and position of supernumerary teeth and their relationship with adjacent permanent teeth but also for identifying the best surgical approach for minimizing damage to adjacent permanent tooth roots and surrounding tissue.

In both non‐CCD and CCD cases, some case reports have described the 3D evaluation of supernumerary teeth for surgical treatment planning.^26^, ^27^ However, few studies have evaluated and summarized the morphology and position of supernumerary teeth.

Moreover, there has been no detailed investigation on supernumerary teeth in patients with CCD using 3D imaging techniques. Therefore, the purpose of this study was to elucidate the morphology and position of supernumerary teeth using 3D imaging techniques.

## 2 MATERIALS AND METHODS

### 2.1 Subjects

The individuals who were clinically diagnosed by specialists as having CCD and treated at the Tokyo Medical and Dental University (TMDU) Dental Hospital within the last 20 years after CBCT had been put to practical use in the dental field were subjected. First, the patients having no supernumerary teeth were excluded. To evaluate the dental root length more accurately, a lower age limit of the average age (15 years old) of dental root completion of the permanent teeth was applied.^28^ Among them, the patients who required CBCT for diagnosis and orthodontic treatment planning were selected. In addition, to avoid any bias for evaluation of the morphology and position of impacted supernumerary teeth in CCD, only those patients who had never received either orthodontic treatment, extraction of primary, permanent or supernumerary teeth, or fenestration‐traction of impacted permanent teeth, which were influenced the development, position, and direction of supernumerary and permanent teeth, at the time of examination were selected. Finally, five Japanese individuals (male, 3; female, 2; age, 15.0‐25.4 years; mean age, 18.2 ± 3.9 years) were included in this study (Table 1). The patients were fully informed of the purpose and risks of CBCT. Precise clinical examination for orthodontic treatment was performed in each case, and stature was evaluated on the basis of age‐ and sex‐matched Japanese norms.^29^ In each case, sex, age at the time of CBCT, systemic findings (ie, clavicular hypoplasia, anterior fontanel patency, and short stature), hereditary characteristics, and the number of supernumerary and unerupted permanent teeth (including wisdom teeth) were listed (Table 1). Informed consent was obtained from the patients and their parents. This study followed the tenets of the Helsinki Declaration. This study protocol was reviewed and approved by the ethics committee of Tokyo Medical and Dental University (approval #D2014‐002).

**Table 1:** Characteristics of the five individuals with cleidocranial dysplasia included in the present study

	Sex	Age	Abnormal suture	Abnormal clavicle	Short stature	Hereditary characteristics	Number of supernumerary teeth	Number of unerupted permanent teeth
Case 1	M	16 Y 1 M	+	+	+ (<‐1SD)	Sporadic	15	20
Case 2	F	15 Y 6 M	+	+	+ (<‐1SD)	Sporadic	10	21
Case 3	M	25 Y 5 M	+	+	−	Sporadic	7	18
Case 4	M	18 Y 11 M	+	+	+ (<‐1SD)	Familial	6	20
Case 5	F	15 Y 0 M	−	+	+ (<‐1SD)	Familial	1	10

*Note*: “Age” denotes the time of cone‐beam computed tomography (CBCT). “Abnormal suture” denotes open or delayed closure of suture. “Abnormal clavicle” denotes hypoplastic or aplastic clavicles. Body height values that were over one SD lower than the age‐ and sex‐matched Japanese norms^29^ are highlighted as <−1 SD.

Abbreviations: F, female; M, male.

### 2.2 Reconstruction of 3D images

**Figure 1:**
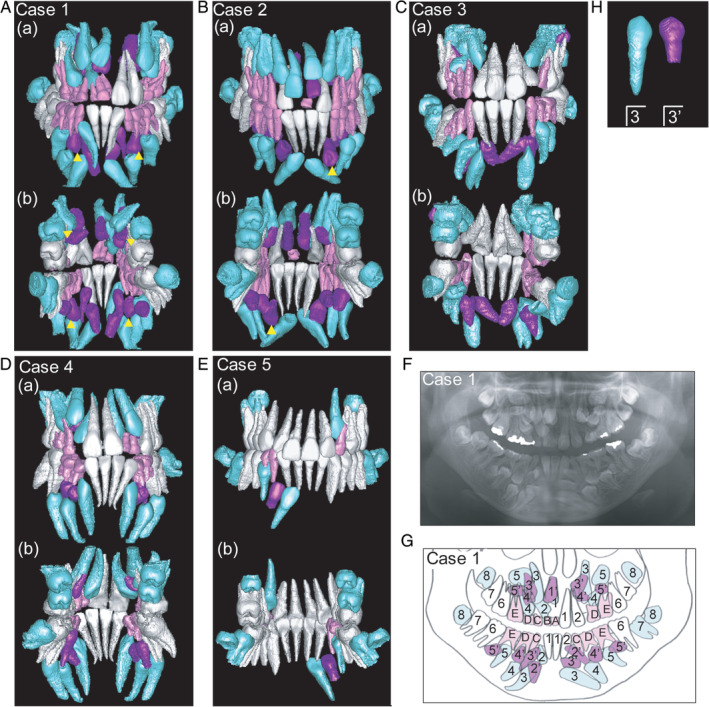
A‐E, Three‐dimensional images derived from cone‐beam computed tomography data of teeth in Cases 1 to 5. (a) Labial and (b) lingual side views. Supernumerary teeth indicating an inverse direction are shown by yellow arrows (△). F, Panoramic radiograph of Case 1. G, Trace of panoramic radiograph of Case 1. Primary, erupted permanent, unerupted permanent, and supernumerary teeth are shown in pink, white, light blue, and purple, respectively. H, Mandibular left canine and its supernumerary teeth in Case 2. Tooth formulas of supernumerary teeth are indicated by adding a comma next to those of the corresponding adjacent permanent teeth

As part of the pretreatment examination, CBCT (Finecube; Yoshida Dental MFG. Co., Tokyo, Japan) and panoramic and intraoral photographs were acquired and analyzed for diagnosis and orthodontic treatment planning. CBCT images of maxillary and mandibular dentoalveolar regions were acquired using the following settings: normal mode (16.8 seconds; 4.10 mGy; 90 kV; and 4 mA); slice thickness of the axial image, 0.147 mm; field of view, 81 × 74 mm; and voxel size, 0.146 mm. These digital axial images were converted to digital imaging and communications in medicine (DICOM) data and reconstructed into 3D images using Simplant OMS (Materialize Dental Japan, Tokyo, Japan). The maxillary and mandibular alveolar bone around the teeth was digitally removed, and the teeth were separated from the whole images by threshold‐based segmentation on the basis of differences in permeability to X‐rays (Figure 1A‐E).^30^, ^31^, ^32^, ^33^, ^34^ Panoramic radiographs were traced, after which primary, erupted permanent, unerupted permanent, and supernumerary teeth were classified and color coded (Figure 1F, G).

### 2.3 Definition of supernumerary tooth

Typically, a supernumerary tooth is identified on the basis of its proximity to the adjacent permanent tooth and distinguished from the permanent tooth by its position, inclination, crown shape, and root formation on panoramic radiographs. In this study, to eliminate any ambiguity in identifying impacted teeth (unerupted permanent teeth and supernumerary teeth) those were in close proximity, impacted teeth with longer and shorter dental roots were defined as permanent and supernumerary teeth, respectively. In addition, permanent teeth positioned closest to their corresponding supernumerary teeth were defined as “adjacent permanent teeth” (including unerupted permanent teeth). Proximity of supernumerary teeth to their corresponding adjacent permanent teeth was confirmed by measurement in three dimensions. The tooth formulas of supernumerary teeth were indicated by adding a comma next to those of their adjacent permanent teeth (Figure 1H).

### 2.4 Evaluation items for supernumerary teeth

#### 2.4.1 Number of cusps and dental roots

**Figure 2:**
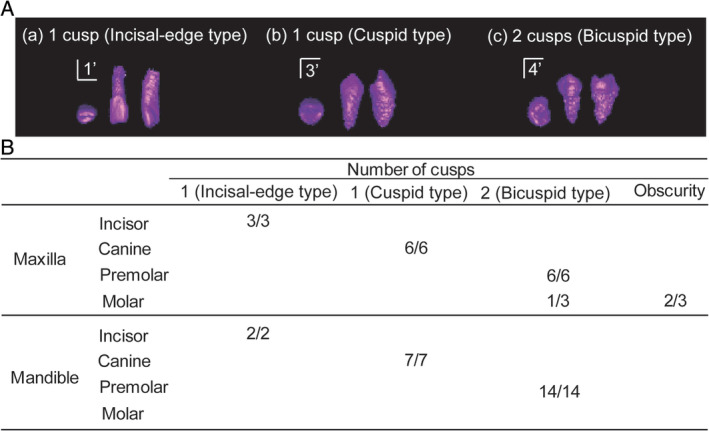
A, Examples of supernumerary teeth with incisal‐edge and cusped types of single‐cusp crowns and the bicuspid type of double cusp crowns. Occlusal surfaces and front and lateral views of supernumerary teeth of the maxillary left central incisor in Case 2 (a) and mandibular left canine (b) and first premolar (c) in Case 3. B, Classification of supernumerary teeth (of all patients) in each region on the basis of number of cusps. The denominator denotes the number of supernumerary teeth in each region, and the numerator denotes the number of supernumerary teeth classified by the number of cusps in each region

In all cases, the locations of supernumerary teeth were classified into eight regions: anterior, canine, premolar, and molar regions in either maxilla or mandible. At each region, the number of cusps and dental roots of supernumerary teeth was determined using CBCT images, respectively. The number of cusps was determined to be one (single‐cusped type) or two (bicuspid type). Single‐cusped supernumerary teeth were further classified on the basis of morphology into incisal‐edge and cuspid (pointed cusp) type teeth (Figure 2A).

#### 2.4.2 Comparison of size between supernumerary and adjacent permanent teeth

For determining the crown and dental‐root lengths, the crown mesiodistal and buccolingual diameters of all supernumerary and adjacent permanent teeth (78 in total) were measured using the Simplant OMS software (Materialize Dental Japan). According to the previous report, crown length was defined from the mid‐point of the cervical reference line, which joined the labial and palatal cementoenamel junction (CEJ), to the highest point of the crown, that is, its cusp tip or incisal edge. Root length was defined from the midpoint of the cervical reference line to the root apex.^35^ The supernumerary and adjacent permanent tooth groups were then compared on the basis of these measurements. In relation to root length, seven teeth that had roots extending out of the imaging range, including maxillary and mandible bilateral canines in Case 1, maxillary left third molar in Case 3, and maxillary bilateral canines in Case 4, were excluded from comparative analysis. In addition, the crown mesiodistal diameter of each supernumerary and adjacent permanent tooth, excluding the maxillary third molar and its corresponding supernumerary teeth in Case 3, was compared with the Japanese norm by *z*‐score analysis ([measurement‐Japanese normal value]/SD). The Japanese normal values for crown mesiodistal diameter were obtained from the report by Otsubo.^36^

#### 2.4.3 Positional relationship between supernumerary and adjacent permanent teeth

Upon setting up reference planes for examining the 3D positions of permanent and supernumerary teeth, the 3D position of each tooth was determined by measuring the distance from each plane to the central part of its occlusal surface (incisal center for teeth in the anterior region; pointed cusp for those in the canine region; and the center of the central groove for those in the premolar and molar regions). The occlusal plane passed through three points, that is, the central fossae of the occlusal surface of the right and left first molars and incisal mid‐line regions of central incisors on both sides. The sagittal plane was perpendicular to the occlusal plane, passing through the mid‐point of a line connecting the central fossae of the occlusal surface of the right and left first molars and the midline region of central incisors on both sides. The frontal plane was perpendicular to the sagittal plane, passing through a point at the central incisal edge of the midline region. The positional relationship between supernumerary and adjacent permanent teeth was analyzed in the coronal and apical, mesial and distal, and labial and lingual sides.

#### 2.4.4 Direction of supernumerary teeth

Tooth axis was defined as a straight line connecting the central part of the occlusal surface as described above and the apex. The direction of supernumerary and unerupted permanent teeth was analyzed by considering positive and negative inclination angles of the tooth axis against the occlusal plane to indicate the normal or inverse direction, respectively.

### 2.5 Measurement errors

To identify measurement errors and determine reproducibility, all measurements of randomly selected subjects were repeated at least 1 week apart by a single investigator. Systematic errors were confirmed by the paired *t* test for comparison between two sets of measurements. In the present study, there were no significant differences between the two sets of measurements, and their interclass correlation coefficients ranged from 0.981 to 0.999. Additionally, random errors were estimated using Dahlberg's formula,^37^ and the values varied from 0.01 to 0.79 mm for linear measurements.

### 2.6 Statistical analysis

All statistical analyses were performed using the SPSS software (ver. 13.0 for Windows; SPSS, Inc., Chicago, Illinois). Wilcoxon's signed‐rank test was used for statistical analysis of significance (*P* < .05).

## 3 RESULTS

### 3.1 Systematic conditions and complications

The clinical and hereditary characteristics of the five subjects are shown in Table 1. All subjects showed patent skull sutures and abnormal clavicles, and their body height values were over one SD lower than previously reported age‐ and sex‐matched Japanese norms (except in Case 3).^34^ With regard to hereditary characteristics, Cases 1, 2, and 3 had sporadic CCD, whereas Cases 4 and 5 had familial CCD.

### 3.2 Number and location of supernumerary teeth

All subjects showed supernumerary and unerupted permanent teeth (Table 1). The number of supernumerary and unerupted permanent teeth ranged from 1 to 15 (average, 7.8) and 10 to 21 (average, 17.8), respectively. There was no instance of a single permanent tooth bearing multiple supernumerary teeth. Three supernumerary teeth in the molar region were observed only in Case 3; these teeth were positioned above the bilateral maxillary first molars and on the labial side of the maxillary third molar (Figure 1C). The frequency of these teeth was the highest in the mandibular premolar region (14/39; 35.9%), followed by the mandibular canine (7/39; 17.9%), maxillary canine (6/39; 15.4%), and maxillary premolar (6/39; 15.4%) regions. No supernumerary teeth were observed in the mandibular molar region.

### 3.3 Evaluation of number of cusps and dental roots

Upon classifying supernumerary teeth on the basis of number of cusps, teeth with incisal‐edge, cuspid‐ (pointed cusp), and bicuspid‐type crowns were observed in the incisor, canine, and premolar regions, respectively (Figure 2B). Three supernumerary teeth in the molar region exhibited were microdontia apparently; therefore, the crown morphology of two of these teeth was obscure, and it was not possible to identify the number of cusps. None of the teeth exhibited more than two cusps. All of the evaluated supernumerary teeth were single‐rooted (19/39; 48.7%) except for those with roots that were too short and small to be distinguished.

### 3.4 Comparison of size between supernumerary and adjacent permanent teeth

**Figure 3:**
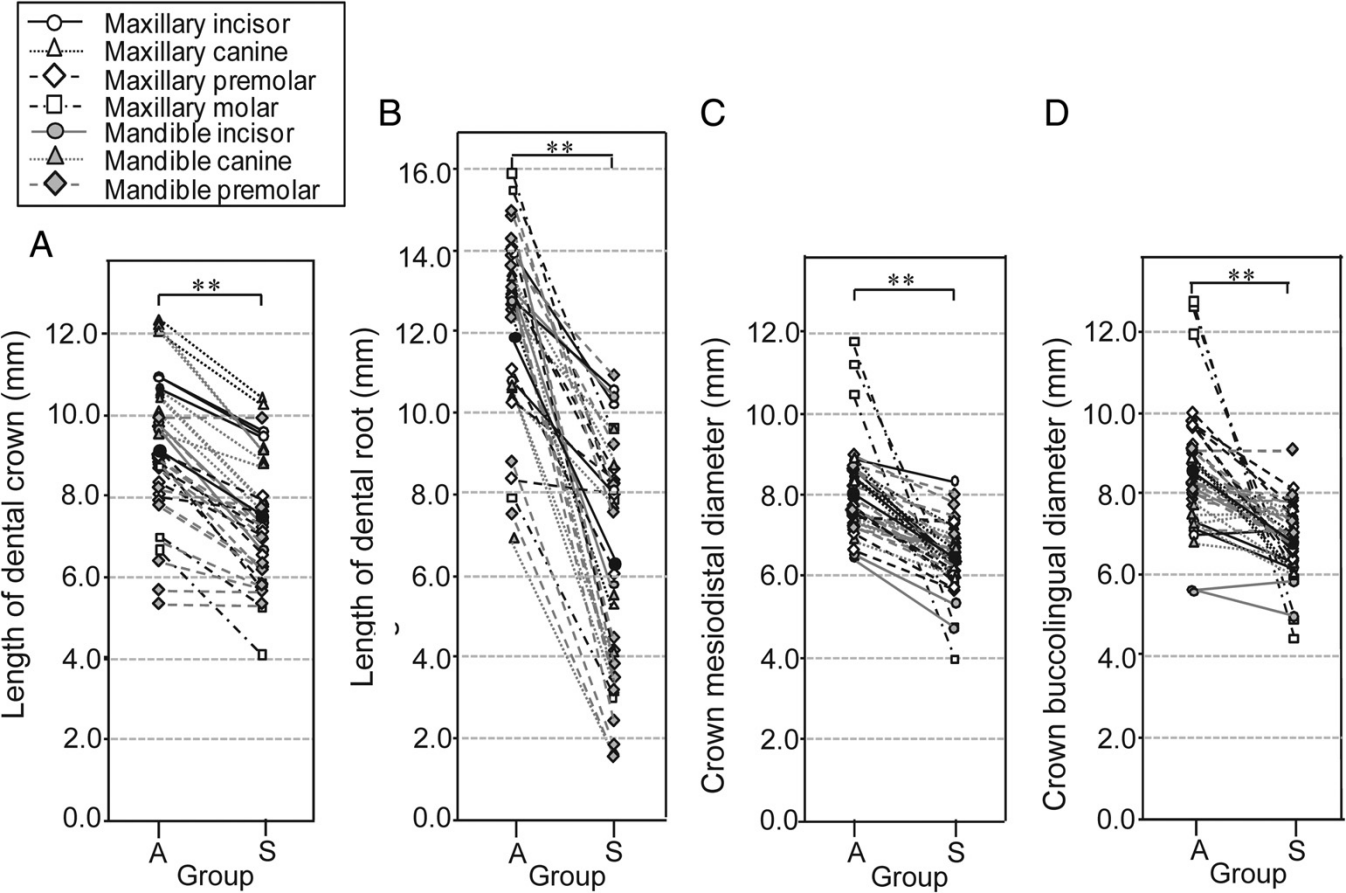
Comparison of size between supernumerary and adjacent permanent teeth on the basis of crown A, and dental root B, lengths and crown mesiodistal C, and buccolingual D, diameters. White and gray (maxilla and mandible, respectively) circles, triangles, and rhombuses denote incisors, canines, and premolars, respectively, and their corresponding supernumerary teeth. White squares denote molars and their corresponding supernumerary teeth in the maxilla. A, adjacent permanent tooth group; S, supernumerary tooth group. *Intergroup statistical differences (^*^ *P* < .05, ^**^ *P* < .01; Wilcoxon's signed‐rank test)

The results of the size comparison between supernumerary and adjacent permanent teeth at each region (Figure 3) demonstrated that supernumerary teeth possessed significantly shorter crown and dental‐root lengths and smaller crown mesiodistal and buccolingual diameters than adjacent permanent teeth (*P* < .01). Comparison of crown mesiodistal diameters of supernumerary and adjacent permanent teeth with the Japanese normal values^36^ yielded average *z*‐scores of −1.95 and + 1.30, respectively. In anterior, canine, premolar, and molar regions, average *z*‐scores of supernumerary teeth resulted in −1.57, −1.70, −1.11 and − 10.1, respectively, and the value in molar region were especially small.

### 3.5 Positional relationship between supernumerary and adjacent permanent teeth

**Figure 4:**
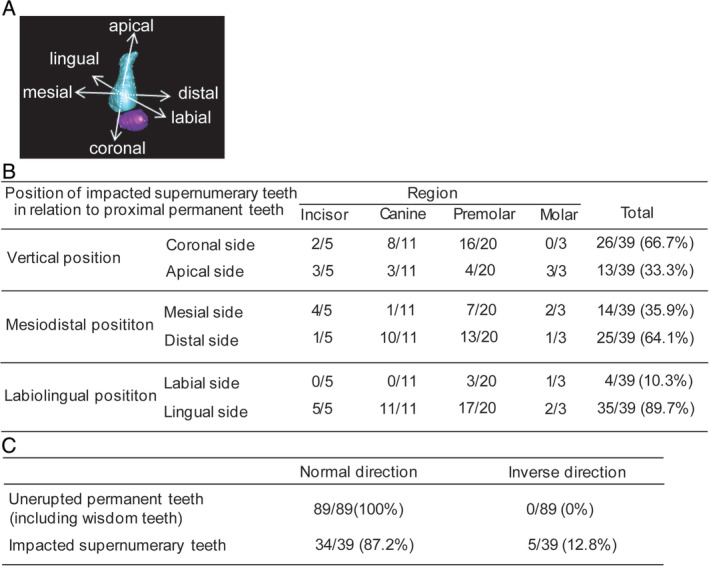
A, Definition of the positional relationship of a supernumerary tooth with an adjacent permanent tooth. An unerupted permanent tooth (maxillary right second premolar in Case 1) and its corresponding supernumerary tooth are shown in light blue and purple, respectively. B, Vertical, mesiodistal, and labiolingual positions of supernumerary teeth in relation to adjacent permanent teeth by region. The denominator denotes the number of supernumerary teeth in each region, and the numerator denotes the number of supernumerary teeth classified by position in each region. C, Direction of supernumerary and adjacent permanent teeth. Inclination angles of the tooth axis against the occlusal plane were positive and negative, indicating the normal and inverse directions, respectively. The denominator denotes the total number of unerupted permanent/supernumerary teeth in all cases, and the numerator denotes the number of teeth indicating the normal/inverse direction

The positional relationship between supernumerary and adjacent permanent teeth was demonstrated according to region (ie, vertical, mesiodistal, and labiolingual positions) (Figure 4A). In canine and premolar regions, supernumerary teeth showed a tendency to be positioned on the coronal (8/11; 72.7% and 16/20; 80.0%, respectively) and distal (10/11; 90.9% and 13/20; 65.0%, respectively) sides. In incisor, canine, and premolar regions, they tended to be positioned on the lingual side (5/5; 100.0%, 11/11; 100.0% and 17/20; 85.0%, respectively). Relative to their corresponding adjacent permanent teeth, 66.7% (26/39) of supernumerary teeth were positioned vertically on the coronal side; 64.1% (25/39) in the mesiodistal direction on the distal side; and 89.7% (35/39) in the labiolingual direction on the lingual side (Figure 4B).

The analysis of the direction revealed positive inclination angles of the tooth axis against the occlusal plane in all adjacent permanent teeth, indicating the normal direction (Figure 4C). In 12.8% (5/39) of the evaluated supernumerary teeth, the inclination angles of the tooth axis against the occlusal plane were negative, indicating the inverse direction. These five teeth—four in Case 1 and one in Case 2—exhibited the inverse direction and are indicated by yellow arrows in 3D images (Figure 1A, B).

## 4 DISCUSSION

The present study is the first to analyze the 3D position, morphology, and size of supernumerary teeth in patients with CCD. The results of relationship analyses between supernumerary and adjacent permanent teeth revealed that, at sites other than the molar region, supernumerary teeth were positioned on the lingual and distal sides and that supernumerary teeth resembled their adjacent permanent teeth in terms of morphology, but the size was smaller than that of the adjacent permanent teeth.

Supernumerary teeth are commonly distinguished from adjacent permanent teeth on panoramic radiographs by the position, inclination, crown shape, and the root formation.^11^ The present study used 3D images to distinguish supernumerary teeth from adjacent permanent teeth on the basis of dental root length without ambiguity. Among the subjects with CCD included in this study, supernumerary teeth existed as pairs with adjacent permanent teeth. Although supernumerary teeth generally have a tendency towards microdontia,^19^, ^20^, ^21^, ^22^ the analysis of crown morphology and size in anterior, canine, and premolar teeth in 3D images revealed that the supernumerary teeth resemble the adjacent permanent teeth in patients with CCD. Because dental root length is an approximate indication of the stage of odontogenesis,^38^ we considered teeth with relatively longer dental roots to be adjacent permanent teeth and those with shorter dental roots to be supernumerary teeth (Figure 1H). The present results on differentiation of supernumerary and adjacent permanent teeth on the basis of the above definition of supernumerary teeth were consistent with those obtained by the conventional method of differentiation using panoramic radiographs (Figure 1F, G). Therefore, our definition of supernumerary teeth on the basis of dental root length can be considered appropriate.

In the present study, the frequency of supernumerary teeth was the highest in the mandibular premolar region and the results correspond with those of previous reports.^9^, ^12^, ^39^ Supernumerary and unerupted permanent teeth were more commonly observed in the anterior, canine, and premolar regions of the maxilla and mandible. Moreover, in almost all cases, supernumerary teeth appeared to be positioned between primary and permanent teeth, which suggested that the presence of supernumerary teeth might have an influence on the eruption of permanent teeth. However, Case 5 exhibited few supernumerary teeth but many unerupted permanent teeth, which could probably be explained by delayed or arrested root resorption of primary teeth.^39^

Previous studies have classified the morphology of supernumerary teeth on the basis of appearance.^19^, ^20^, ^21^, ^22^ In the present study, to eliminate bias due to subjectivity in judging the morphology of supernumerary teeth, the similarity of supernumerary teeth to the corresponding adjacent permanent teeth was evaluated on the basis of number of cusps in 3D images. The results showed that supernumerary teeth possessed incisal‐edge, pointed‐cusp, and bicuspid‐type crowns in the anterior, canine, and premolar regions, respectively. Thus, supernumerary teeth exhibited apparent similarities with adjacent permanent teeth in terms of crown morphology, suggesting a relationship with their location (ie, region specificity) (Figure 2B). A previous finding showing that supernumerary teeth resemble their corresponding adjacent permanent teeth^40^ is supported by the present findings on supernumerary teeth in the anterior, canine and premolar regions.

The results of the measurement of 39 supernumerary teeth and their corresponding adjacent permanent teeth in 3D images revealed that the sizes of the supernumerary teeth were smaller than adjacent permanent teeth (Figure 3). A previous study showed abnormalities in tooth morphology in patients with CCD,^12^ which were presumed to be related to inadequate space and arrested eruption, that is, spatial crowding in the alveolar bone might influence the development of dental crowns and roots.^39^

With regard to positional relationships between supernumerary and adjacent permanent teeth, the present findings revealed a tendency of supernumerary teeth to be positioned at the coronal, distal, or lingual side of adjacent permanent teeth other than the molar region (Figure 4B). As for the direction, 12.8% (5/39) of the supernumerary teeth exhibited the inverse direction (Figure 4C). Commonly, inverse impacted teeth are frequently observed in mesiodens, and an inverse impacted third molar is sometimes observed.^41^, ^42^ Further studies are necessary to determine whether the direction of supernumerary teeth is already abnormal at the tooth‐germ formation stage or such an abnormality occurs in the development process as a result of insufficient space for tooth formation.

Although the etiology of supernumerary teeth in CCD is still unclear, overproliferation or prolonged survival of dental laminar epithelial cells has been suggested to cause supernumerary teeth.^40^, ^43^, ^44^ Another possible origin for supernumerary units, according to the tooth‐germ dichotomy theory, is division of dental lamina during odontogenesis, resulting in multiple teeth.^44^, ^45^ In the present study, the crown mesiodistal diameter of adjacent permanent teeth was not smaller than the Japanese average value, as demonstrated by the results of *z*‐score analysis. Theoretically, when a single tooth germ is divided into multiple tooth germs, both supernumerary and adjacent permanent teeth should be small.^46^ Therefore, the present findings are not consistent with the tooth‐germ dichotomy theory.

The molar‐adjacent supernumerary teeth in Case 3 were microdontia, which were apparently small and obscure morphologically, and one of these three teeth was positioned on the buccal side of its adjacent permanent tooth (Figure 1C, 2B). The anterior, canine, and premolar regions are characterized by formation of primary teeth that belong to different tooth species and are later replaced by a single set of permanent teeth. Primary teeth are initiated from the primary dental lamina and replacement teeth from the successional dental lamina on the lingual and distal side of primary teeth.^43^, ^47^

If one considers that supernumerary teeth in patients with CCD develop from the successional lamina from primary teeth, just as permanent teeth do, it could explain why supernumerary teeth form on the coronal, lingual, and distal sides in the anterior, canine, and premolar regions, whereas the supernumerary teeth in the molar region, which did not have replacement teeth, might develop by different mechanisms. This is thought to be one reason for supernumerary teeth being markedly small in the molar region.

The relationships among dental lamina, enamel organs, and gene‐expression patterns in mesenchymal tissues determine the region of formation of each tooth species.^48^, ^49^ The protein RUNX2 is associated with osteoblast regulation and epithelial‐mesenchymal interaction in tooth development and morphogenesis.^50^, ^51^, ^52^ Although supernumerary teeth can develop as a result of a breakdown in mechanisms associated with RUNX2 haploinsufficiency, the transcription factor seems to work in regard to regulation of tooth species (ie, morphology) peculiar to regions, to a certain extent. The pathological process of development of supernumerary teeth in CCD requires further investigation from a molecular‐biological standpoint.

Supernumerary teeth cause serious problems in occlusion, dentition, and mastication. The advent of 3D image analysis has made it possible to obtain information regarding the morphology and positional relationship of impacted supernumerary teeth and their adjacent permanent teeth in patients with CCD, which had been unclear from 2D data derived from panoramic radiographs. The present findings on 3D analysis of supernumerary teeth will be useful in eruption guidance, tooth extraction, and other clinical treatment. They might also help elucidate the pathophysiology of CCD. However, because of the limited number and age of patients, our findings cannot be generalized and the position and the direction of supernumerary teeth at an early stage were not investigated. Analysis of the status of tooth germs in young patients at an early stage might help discern the position, the direction and order of development of supernumerary teeth.

## DISCLOSURES OF INTERESTS

None.
